# Impact of the Liquid Crystal Order of Poly(azomethine-sulfone)s on the Semiconducting Properties

**DOI:** 10.3390/polym14071487

**Published:** 2022-04-06

**Authors:** Oana Dumbravă, Dumitru Popovici, Decebal Vasincu, Ovidiu Popa, Lăcrămioara Ochiuz, Ștefan-Andrei Irimiciuc, Maricel Agop, Anca Negură

**Affiliations:** 1 “Petru Poni” Institute of Macromolecular Chemistry, Gr. Ghica Voda Alley, 41A, 700487 Iasi, Romania; dumbrava.oana@icmpp.ro (O.D.); dumitru.popovici@icmpp.ro (D.P.); 2 Department of Biophysics and Medical Physics, Faculty of Dental Medicine, “Grigore T. Popa” University of Medicine and Pharmacy, 16 University Str., 700115 Iasi, Romania; decebal.vasincu@umfiasi.ro; 3 Department of Emergency Medicine, Faculty of Medicine, “Grigore T. Popa” University of Medicine and Pharmacy, 16 University Str., 700115 Iasi, Romania; ovidiu.popa@umfiasi.ro; 4 Department of Pharmaceutical Sciences, Faculty of Pharmacy, “Grigore T. Popa” University of Medicine and Pharmacy, 16 University Str., 700115 Iasi, Romania; lacramioara.ochiuz@umfiasi.ro; 5 National Institute for Laser, Plasma and Radiation Physics, 409 Atomistilor Street, 077125 Bucharest, Romania; 6 Department of Physics, “Gh. Asachi” Technical University of Iasi, 700050 Iasi, Romania; 7 Romanian Scientists Academy, 54 Splaiul Independentei, 050094 Bucharest, Romania; 8 Faculty of Biology, “Alexandru Ioan Cuza” University of Iasi, 2A Carol Boulevard, 700505 Iasi, Romania; anca.negura@uaic.ro

**Keywords:** poly(azomethine), semiconducting, mesophase, biomedicine, multifractal model

## Abstract

Organic semiconductors are an attractive class of materials with large application in various fields, from optoelectronics to biomedicine. Usually, organic semiconductors have low electrical conductivity, and different routes towards improving said conductivity are being investigated. One such method is to increase their ordering degree, which not only improves electrical conduction but promotes cell growth, adhesion, and proliferation at the polymer–tissue interface. The current paper proposes a mathematical model for understanding the influence of the ordering state on the electrical properties of the organic semiconductors. To this end, a series of aromatic poly(azomethine)s were prepared as thin films in both amorphous and ordered states, and their supramolecular and electrical properties were analyzed by polarized light microscopy and surface type cells, respectively. Furthermore, the film surface characteristics were investigated by atomic force microscopy. It was established that the manufacture of thin films from mesophase state induced an electrical conductivity improvement of one order of magnitude. A mathematical model was developed in the framework of a multifractal theory of motion in its Schrodinger representation. The model used the order degree of the thin films as a fractality measure of the physical system’s representation in the multifractal space. It proposed two types of conductivity, which manifest at different ranges of fractalization degrees. The mathematical predictions were found to be in line with the empirical data.

## 1. Introduction

Organic semiconductors constitute a class of materials intensely studied in the last decades as suitable candidates to replace inorganic ones because of their numerous advantages, such as light weight, mechanical flexibility, and ability to be processed from solution at low temperatures, resulting in low-cost production [1]. Their successful use in biomedicine increased researchers’ interest in these materials, which offer the advantage of electrical control over a large range of physical and chemical properties, being considered a new generation of “smart” biomaterials [2,3,4,5]. They were investigated to be applied as scaffolds in tissue engineering [2], building of biosensors [3], biointerfacing, and implantable electronics and bionics [4]. The native electrical properties of the muscles, heart, bone, and brain allow external electric stimulation to affect various healing processes [5]. As an example, numerous studies reported the potential of semiconducting polymers for building implantable brain machines for treating neurologic diseases such as epilepsy, chronic pain, Parkinson’s disease, and other disfunctions by identifying the signaling network and delivering neural impulses to the brain area [5]. It was demonstrated that the specific response could be modulated by surface characteristics such as roughness, topography, chemistry and charge [2]. Furthermore, the soft interface of organic semiconductors favors interaction with living tissues, and good nanostructuring can regulate cellular behavior, including adhesion, growth, migration, and regeneration of damaged tissues [5]. Compared with their inorganic counterparts, the low modulus of organic materials avoids mechanical trauma and chronic inflammation at the interface [2,3,4,5]. Among organic semiconductors, aromatic poly(azomethine)s are especially promising because of their good charge carrier mobility and facile preparation routes, with high yields and no side reactions, leading to products of high purity [6]. The reversible nature of azomethine linkage makes them benign candidates for bioapplications, allowing disintegration into biocompatible products [7]. Their good charge carrier mobility derives from the extended electronic conjugation supported by the azomethine (N=C) bond (also known as imine or Schiff base), which is isoelectronic with the vinylene bond (C=C) [8]. An important property of the poly(azomethine)s, which has been little exploited in the semiconductor domain, is their ability to self-order into the melt state, forming thermotropic mesophases [9,10,11]. This is possible because of the high conjugation of the azomethine units assuring the charge separation, which further favors intermolecular forces that stabilize the mesophase state. Thus, on a hand, the ordered phase assures better electron mobility by favoring the electron jumping among the molecules and on the other hand by creating continuous films [12]. A study on a series of poly(azomethine-sulfones)s indicated that the presence of sulfone and isopropyl units into a polyazomethine backbone is beneficial for promoting liquid crystalline and semiconducting properties [13,14]. Moreover, it was demonstrated that this chemical design was favorable for obtaining nanostructured ordered thin films via a simple thermal treatment consisting of quenching from the mesophase state. In this light, the present study proposes developing a mathematical model in a multifractal space to understand the influence of the ordering degree of mesophases on the semiconducting properties. To this end, thin poly(azomethine sulfone)s films were prepared by casting from solution, and then they were thermally treated from the mesophase state. The supramolecular organization of the films, thermally treated and pristine, was investigated by polarized light microscopy, and their semiconducting properties were determined with a surface type cell.

The analysis of the fractal characteristics proved to be a useful asset to appreciate the microstructure and the interphase interactions in various systems. Thus, the calculation of the fractal dimension was used to assess: the interfacial microstructure and π–π stacking in composite systems of conjugated polyimides with multiwalled carbon nanotubes [15], microstructure evolution effect on the thermal conductivity of low density polyethylene and boron nitride nanosheets [16], growth of titanium oxide quantum dots into polyaniline semiconductor [17], the relationship between structure evolution and electrical conductivity of polyurethanes doped with carbon nanotubes [18]. All these data indicate the successful use of fractal characteristics for evaluation of the influence of microstructure on different properties, such as electrical ones. In this light, the paper proposes a mathematical model in a multifractal space to evaluate the microstructure—electrical conductivity relationship, as follows. The use of a multifractal representation when investigating the physical and chemical properties of polymeric materials has been reported by our group in the past few years, with the main focus being on drug release mechanisms at various scale resolutions [19,20,21]. The multifractal model is dynamical and based on Scale Relativity Theory, and it works on the underlying hypothesis that the entities of any complex system move on continuous and nondifferential curves, named fractal curves, i.e., three dimensional fractured lines, the nonlinearity of which is dependent on and proportional with the number of interactions within the system. Such representation allows the utilization of the fractalization degree as a measure of system complexity and physical quantities, characterizing both the nature of the system (degrees of order-disorder) and the system evolution. This approach allows the use of fractal functions dependent both on spatiotemporal coordinates and resolution scales. This means that the model can look at the evolution of the system and its properties simultaneously by using appropriate choices for the scale resolutions and fractalization degree. Furthermore, complex systems (here, poly(azomethine sulfone) polymer) can be considered as media without interaction between their components. Dynamics in the hydrodynamic multifractal representation were used as a benchmark for the study of the transient physical properties of the polymeric material.

## 2. Materials and Methods

*Thin film preparation*

A series of poly(azomethine sulfones)s were synthetized according to [13]. They were solubilized in DMF to give 1% solutions, which were casted on glass lamella and incubated for 72 h at 50 °C to give thin films. Ordered thin films were prepared by thermal treatment of the thin films on a hot stage under polarized light, heated at 10 °C/min up to the mesophase state, annealed for 30 min, and quenched at room temperature. Thus, two series of films were prepared. The untreated films were abbreviated **P1**–**P4**, and the treated films, **P1** *–**P4** *. The thickness of the films before thermal treatment was around 1.6 μm (P1: 1.84 μm, P2: 1.58 μm; P3: 1.05 μm, P4: 0.38 μm).

*Equipment*

Thermal treatment of the films was realized by continuous observation of the thermotropic behavior of the films with an Olympus BH-polarized light microscope (Olympus, Tokyo, Japan)equipped with a THMS 600/HSF9I hot stage and a Linkam temperature control system. For all measurements, a 40× objective was used. The eyepiece had 10× magnification.

The thickness of the untreated films was determined by Fizeau’s method for fringes of equal thickness, using an interferential microscope MII-4(JSC “LOMO”, St. Petersburg, Russia).

The electrical conductivity of both the treated and untreated thin films was measured on a Keithley Model 6517 electrometer(Tektronix, Beaverton, Oregon, United States). The measurements were taken in triplicate, and the mean values are given. The resistivity of the polymeric coating was measured by applying a two-point technique. This method consisted of voltage drop measurements across the thin film involving a constant current passing through it.

The topography of the film surface was assessed from images collected in semi-contact mode with a Solver PRO-M, NT-MDT atomic force microscope (AFM) (NT-MDT Spectrum Instruments, Moscow, Russia), using a Zelenograd NSG10 cantilever (NT-MDT Rusia), with resonant frequency 330 KHz and force constant 26 n/m. The Nova V.1443 software was used for recording and analyzing the AFM topographic images. The arithmetic average roughness (Ra) was determined for all explored areas using the definition expressed by: $Ra=\frac{1}{n}\overset{n}{\underset{i=1}{\sum }}\left[Zi\right]$, where Zi is the value of the tip height in each point of the image over a reference baseline (Z ¼ 0). The images were collected on four different film areas by aleatory “landing” of the cantilever on the film surface and registering data for concentric squares from 0.1 μm × 0.1 μm up to 60 μm × 60 μm. For an accurate comparison of the film surface characteristics, the roughness exponent (RE) was calculated as the slope of roughness versus scan size in a double log plot (Figure S1).

## 3. Results

**Figure 1:**
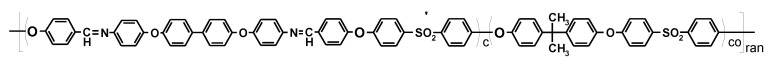
The structure of the poly(azomethine)s **P1**–**P4**. The difference among the four polymers was the different content of mesogenic units: **P1** (100%); **P2** (75%); **P3** (50%); **P4** (25%).

**Figure 2:**
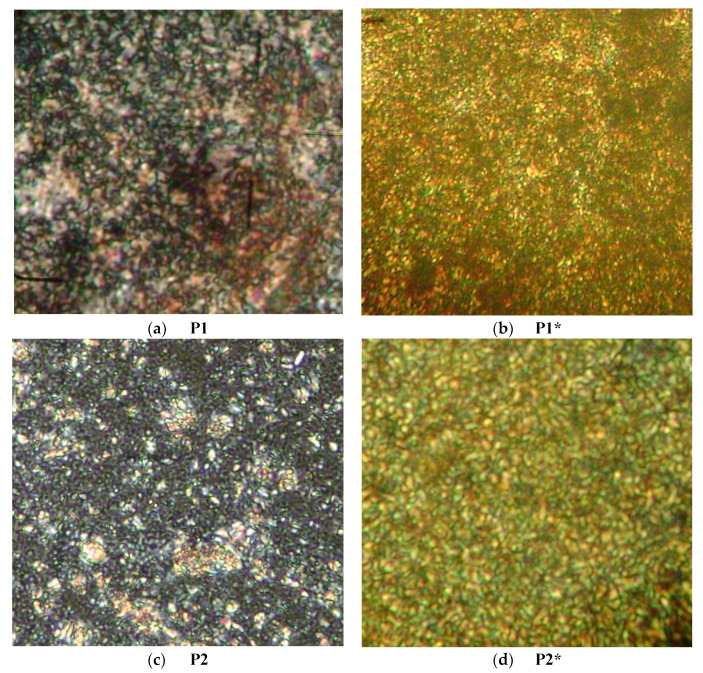

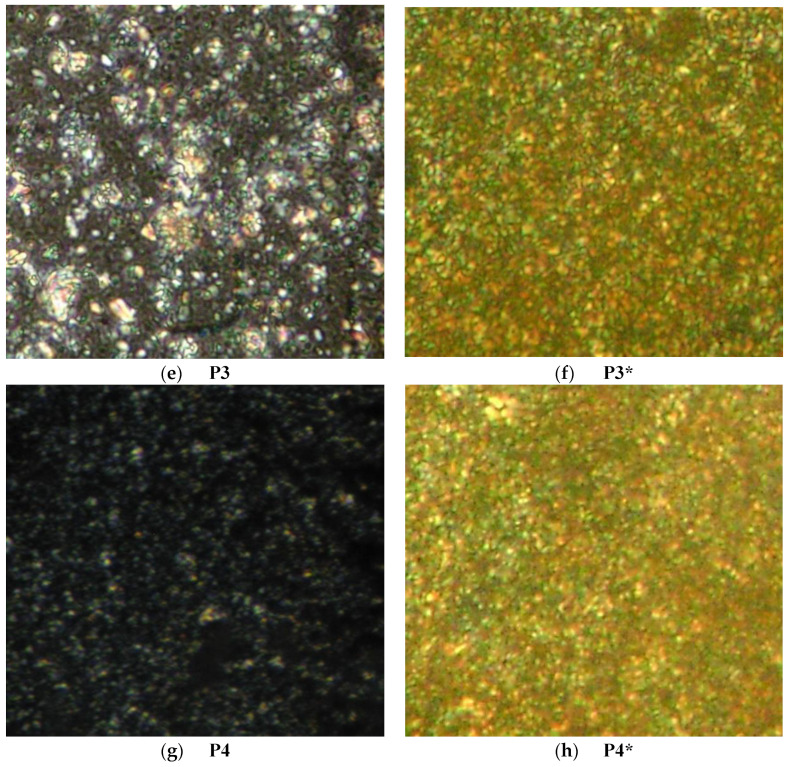
Representative POM images of the treated (**a**–**d**) and untreated (**e**–**h**) thin films. POM images were acquired using the 40× objective, and the eyepiece had 10× magnification, giving a theoretical magnification of 400×.

A series of thin films of aromatic poly(azomethine)s (Figure 1), in ordered state, were prepared by thermal treatment under polarized light. Thin films obtained by casting without any thermal treatment were used as reference in order to evaluate the influence of the ordering on electrical conduction. As can be seen in Figure 2, the ordered films presented intense birefringence, with a fine Schlieren texture, indicating a nematic mesophase in which the azomethine rigid mesogens were preponderantly aligned parallel to a local director [22,23,24,25]. The texture clarity was correlated to the percentage of mesogen in the polymer, with more resolved textures observed for polymers containing 75% (**P2**) or 50% (**P3**) mesogens. Furthermore, the ordered films did not present any cracks or defects, suggesting that continuous ordered films were formed (Figure 2a–d) [26]. The untreated films presented rare birefringent domains into a dark field under polarized light, indicating that the rare ordered domains dispersed into an amorphous one (Figure 2e,f). Moreover, under normal light, it was also seen that the thermal annealing supported the formation of a continuous film without defects, while cracks were present in the untreated film.

**Figure 3:**
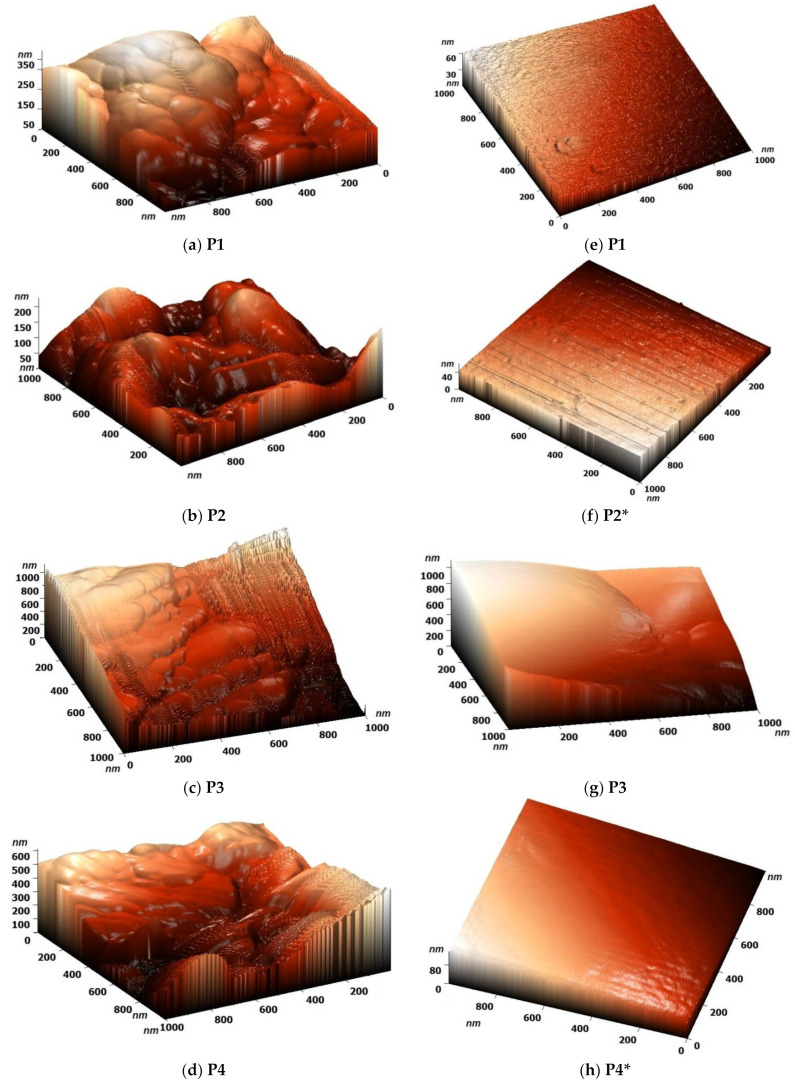

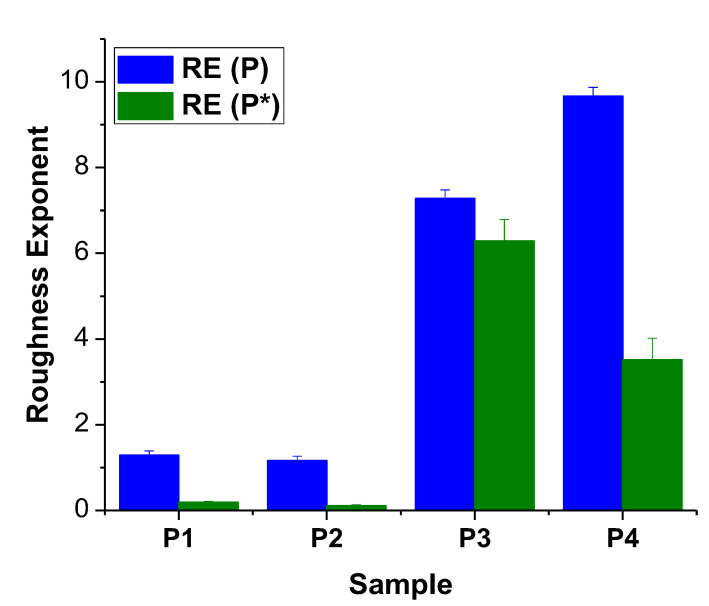
Representative 3D AFM images of the (**a**–**d**) untreated and (**e**–**h**) treated thin films acquired on squares of 1000 × 1000 nm and (**c**) the graphical representation of their roughness exponent (RE (P): roughness exponent of the untreated samples; RE (P*): roughness exponent of the treated samples).

The analysis of the topography of the treated and untreated thin films clearly showed that the thermal annealing transformed the rough films into smoother ones (Figure 3a–h, Table S1), in accordance with the quenching from the molten ordered phase. Moreover, the graphical representation of the roughness exponent [27] of the untreated and treated films undoubtedly indicated that the manufacturing of the films from molten mesophase not only improved the ordered state but was accompanied by an enhancement of the film continuity, which is beneficial for better contact between the organic film and electrodes (Figure S1) [13,28]. Per the AFM images, for the untreated samples, the rough surface was of fractal nature, with edges and broken geometries characterizing the surface of the sample. This translated into an elevated fractalization degree that was further used as reference when developing the model. After treatment, the surface was clearly smoother and thus described by a lower degree of fractalization.

**Figure 4:**
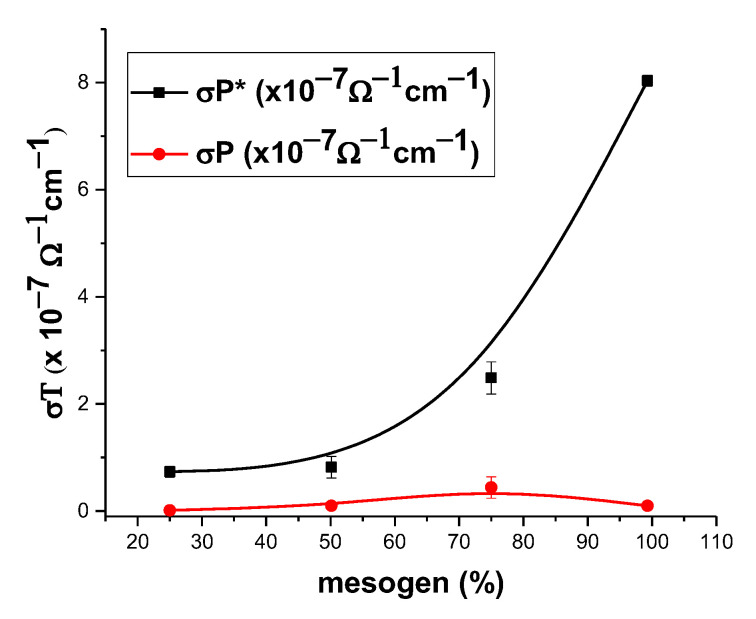
Graphical representation of the electrical conductivity versus percent of azomethine mesogens of polymers for untreated (P) and treated (P*) films.

The measurements of the electrical conductivity (σ) showed values in the range of 10^−7^−10^−8^ Ω^−1^cm^−1^, which are typical values for undoped organic semiconductors [29]. To facilitate easy comparison, they are expressed as x 10^−7^ Ω^−1^cm^−1^. In Figure 4, the electrical conductivity values recorded for the untreated films (coded σP) and those in ordered state (coded σP*) are graphically represented as a function of the percentage of mesogen in the polymer. The values of the electrical conductivity of the untreated films were low, regardless of the percentage of mesogen. A slightly higher value for the polymer **P2**, which contained 75% mesogen, was considered due to the possible formation of cybotactic groups favored by a good balance between rigid azomethine mesogens and flexible sulfone and isopropyl units [30,31]. On the other hand, all the films in ordered state presented significant increases in electrical conductivity, 74 times higher in the case of **P1** with 100% azomethine mesogen. The obvious conclusion is that the ordering of the rigid azomethine units into films favored electron conductivity, most probably because of the close contact of the azomethine mesogens favoring electron jumping [32]. It appears that in the case of **P1**, this effect was particularly effective because of the rigid azomethine chains with high conjugation favoring good longitudinal charge mobility. The longitudinal conduction and the side-jump motion of electrons proved to be an excellent combination for maximizing the electrical conductivity.

**Mathematical model**

To further understand the improvement in conductivity due to thermal treatment of the films into ordered state, a theoretical model was developed. In the description of complex system dynamics through a hydrodynamic multifractal scenario [33,34,35,36], it is possible to find the involvement of the specific multifractal impulse conservation law:

(1) $$\partial_{t}v^{i}+v^{l}\partial_{l}v^{i}=-\partial^{i}Q,\;i=1,2,3$$

and that of the conservation law of the multifractal states density:

(2) $$\partial_{t}\rho +\partial^{l}\left(\rho v^{l}\right)=0$$

where:

(3) $$\partial_{t}=\frac{\partial }{\partial t},\partial_{l}=\frac{\partial }{\partial x^{l}}$$

$$v^{i}=2\lambda {\left(dt\right)}^{\left[\frac{2}{f\left(\alpha \right)}\right]-1}\partial^{i}s,\;u^{i}=\lambda {\left(dt\right)}^{\left[\frac{2}{f\left(\alpha \right)}\right]-1}\partial^{i}\ln\rho$$

$$\rho =\psi \bar{\psi },\;\psi =\sqrt{\rho }e^{is}$$

$$Q=2\lambda^{2}{\left(dt\right)}^{\left[\frac{4}{f\left(\alpha \right)}\right]-2}\frac{\partial_{l}\partial^{l}\sqrt{\rho }}{\sqrt{\rho }}=\frac{u_{i}u^{i}}{2}+\lambda {\left(dt\right)}^{\left[\frac{2}{f\left(\alpha \right)}\right]-1}\partial^{l}u_{l}$$

In the above relations, the given measures have the following physical meanings:

− t is nonmultifractal time, an affine parameter of the movement curves of the entities found in the complex system;
− $x^{l}$ is the multifractal spatial coordinate;
− $v^{i}$ is the velocity field at a differentiable scale resolution;
− $u^{i}$ is the velocity field at a nondifferentiable scale resolution;
− dt is the scale resolution;
− λ is a constant coefficient associated with the multifractal–nonmultifractal scale transition;
− ρ is the state density;
− ψ is the state function with the amplitude $\sqrt{\rho }$ and phase s;
  Q is the scalar specific multifractal potential, which quantifies the multifractalization degree of the movement curves in the complex system;
− $f\left(\alpha \right)$ is the singularity spectrum of order $\alpha =\alpha \left(D_{F}\right)$ where $D_{F}$ is the fractal dimension of the movement curves of the complex system entities. This spectrum allows the identification of universality classes in the complex system dynamics, even when attractors have different aspects, and it also allows the identification of areas in which the dynamics can be characterized by a specific fractal dimension.

Because of their nonlinearity, Equations (1) and (2) admit analytical solutions only in special, particular cases. Such a case is dictated by one-dimensional dynamics of complex system entities through the following:

(4) $$\partial_{t}v+v\partial_{x}v=2\lambda^{2}{\left(dt\right)}^{\left[\frac{4}{f\left(\alpha \right)}\right]-2}\frac{\partial_{xx}\sqrt{\rho }}{\sqrt{\rho }}$$

$$\partial_{t}\rho +\partial_{x}\left(\rho v\right)=0$$

with the initial and boundary constraints:

(5) $$v\left(x,t=0\right)=v_{0},\;\rho \left(x,t=0\right)=\rho_{0}e^{-{\left(\frac{x}{a}\right)}^{2}}$$

$$v\left(x=ct,t\right)=v_{0},\;\rho \left(x=-\infty ,t\right)=\rho \left(x=+\infty ,t\right)=0$$

The following solution is found:

(6) $$v=\frac{v_{0}a^{2}+{\left[\frac{\lambda {\left(dt\right)}^{\left[\frac{2}{f\left(\alpha \right)}\right]-1}}{a}\right]}^{2}xt}{a^{2}+{\left[\frac{\lambda {\left(dt\right)}^{\left[\frac{2}{f\left(\alpha \right)}\right]-1}}{a}t\right]}^{2}}$$

(7) $$\rho =\frac{\pi^{-\frac{1}{2}}}{{\left\{a^{2}+{\left[\frac{\lambda {\left(dt\right)}^{\left[\frac{2}{f\left(\alpha \right)}\right]-1}}{a}t\right]}^{2}\right\}}^{\frac{1}{2}}}\cdot e^{\left\{-\frac{{\left(x-v_{0}t\right)}^{2}}{a^{2}+{\left[\frac{\lambda {\left(dt\right)}^{\left[\frac{2}{f\left(\alpha \right)}\right]-1}}{a}t\right]}^{2}}\right\}}$$

This solution, through the nondimensional variables:

(8) $$\frac{v}{v_{0}}=\bar{v},\;\rho \sqrt{\pi }a=\bar{\;\rho },\;\frac{x}{v_{0}\tau }=\xi ,\;\frac{t}{\tau }=\eta$$

and through the nondimensional parameters:

(9) $$\theta =\frac{\lambda {\left(dt\right)}^{\left[\frac{2}{f\left(\alpha \right)}\right]-1}\tau }{a^{2}},\;\mu =\frac{v_{0}\tau }{a}$$

can be rewritten as:

(10) $$\bar{v}=\frac{1+\theta^{2}\xi \eta }{1+\theta^{2}\eta^{2}}$$

(11) $$\bar{\rho }=\frac{1}{\sqrt{1+\theta^{2}\eta^{2}}}\cdot e^{\left[-\mu^{2}\frac{{\left(\xi -\eta \right)}^{2}}{1+\theta^{2}\eta^{2}}\right]}$$

Through Equation (3), the solutions in Equations (6) and (7) allowed us to construct the following set of variables:

− the velocity field at a nondifferentiable scale:
  (12) $$u=2\lambda {\left(dt\right)}^{\left[\frac{2}{f\left(\alpha \right)}\right]-1}\cdot \frac{\left(x-v_{0}t\right)}{a^{2}+{\left[\frac{\lambda {\left(dt\right)}^{\left[\frac{2}{f\left(\alpha \right)}\right]-1}}{a}t\right]}^{2}}$$
− the specific multifractal force field:
  (13) $$f=-\partial_{x}Q=2\lambda {\left(dt\right)}^{\left[\frac{4}{f\left(\alpha \right)}\right]-2}\cdot \frac{\left(x-v_{0}t\right)}{{\left\{a^{2}+{\left[\frac{\lambda {\left(dt\right)}^{\left[\frac{2}{f\left(\alpha \right)}\right]-1}}{a}t\right]}^{2}\right\}}^{2}}$$

This set of variables employs the notations:

(14) $$\frac{u}{2v_{0}}=\bar{u},\;\frac{f\tau }{2v_{0}}=\bar{f}$$

Considering Equations (8) and (9), they become:

(15) $$\bar{u}=\theta \frac{\xi -\eta }{1+\theta^{2}\eta^{2}}$$

(16) $$\bar{f}=\theta^{2}\frac{\xi -\eta }{{\left(1+\theta^{2}\eta^{2}\right)}^{2}}$$

Then, let us assume the functionality, in nondimensional coordinates, of a relation of the form:

(17) $$\bar{J}=\bar{\sigma }\bar{f}$$

where $\bar{J}$ is a mass current density, $\bar{f}$ is the nondimensional specific multifractal force field, and $\bar{\sigma }$ is a mass conductivity, which then allows us to define the following conductivity types:

− conductivity at differentiable scale resolutions:

(18) $$\bar{\sigma_{D}}=\frac{\bar{\rho }\bar{v}}{f}=\sqrt{1+\theta^{2}\eta^{2}}\frac{1+\theta^{2}\xi \eta }{\theta^{2}\left(\xi -\eta \right)}e^{\left[-\mu^{2}\frac{{\left(\xi -\eta \right)}^{2}}{1+\theta^{2}\eta^{2}}\right]}$$

− conductivity at nondifferentiable scale resolutions:

(19) $$\bar{\sigma_{F}}=\frac{\bar{\rho }\bar{u}}{f}=\sqrt{1+\theta^{2}\eta^{2}}{\left(\frac{\mu }{\theta }\right)}^{2}e^{\left[-\mu^{2}\frac{{\left(\xi -\eta \right)}^{2}}{1+\theta^{2}\eta^{2}}\right]}$$

− conductivity at global scale resolutions:

(20) $$\bar{\sigma }=\frac{\bar{\rho }\left(\bar{v}+i\bar{u}\right)}{f}=\bar{\sigma_{D}}+i\bar{\sigma_{F}}=\sqrt{1+\theta^{2}\eta^{2}}\left[\frac{1+\theta^{2}\xi \eta }{\theta^{2}\left(\xi -\eta \right)}+i{\left(\frac{\mu }{\theta }\right)}^{2}\right]e^{\left[-\mu^{2}\frac{{\left(\xi -\eta \right)}^{2}}{1+\theta^{2}\eta^{2}}\right]}$$

In this context, since the θ parameter is a measure of the multifractality degree, then $\varepsilon =\frac{1}{\theta }$ functions as a measure of ordering degree. Then, the conductivity species in Equations (18)–(20) change as:

− conductivity at differentiable scale resolutions:

(21) $$\bar{\sigma_{D}}=\sqrt{\varepsilon^{2}+\eta^{2}}\frac{\varepsilon^{2}+\xi \eta }{\varepsilon \left(\xi -\eta \right)}e^{\left[-{\left(\mu \varepsilon \right)}^{2}\frac{{\left(\xi -\eta \right)}^{2}}{\varepsilon^{2}+\eta^{2}}\right]}$$

− conductivity at nondifferentiable scale resolutions:

(22) $$\bar{\sigma_{F}}=\sqrt{\varepsilon^{2}+\eta^{2}}\varepsilon \mu^{2}e^{\left[-{\left(\mu \varepsilon \right)}^{2}\frac{{\left(\xi -\eta \right)}^{2}}{\varepsilon^{2}+\eta^{2}}\right]}$$

− conductivity at global scale resolutions:

(23) $$\bar{\sigma }=\sqrt{\varepsilon^{2}+\eta^{2}}\left[\frac{\varepsilon^{2}+\xi \eta }{\varepsilon \left(\xi -\eta \right)}+i\varepsilon \mu^{2}\right]e^{\left[-{\left(\mu \varepsilon \right)}^{2}\frac{{\left(\xi -\eta \right)}^{2}}{\varepsilon^{2}+\eta^{2}}\right]}$$

**Figure 5:**
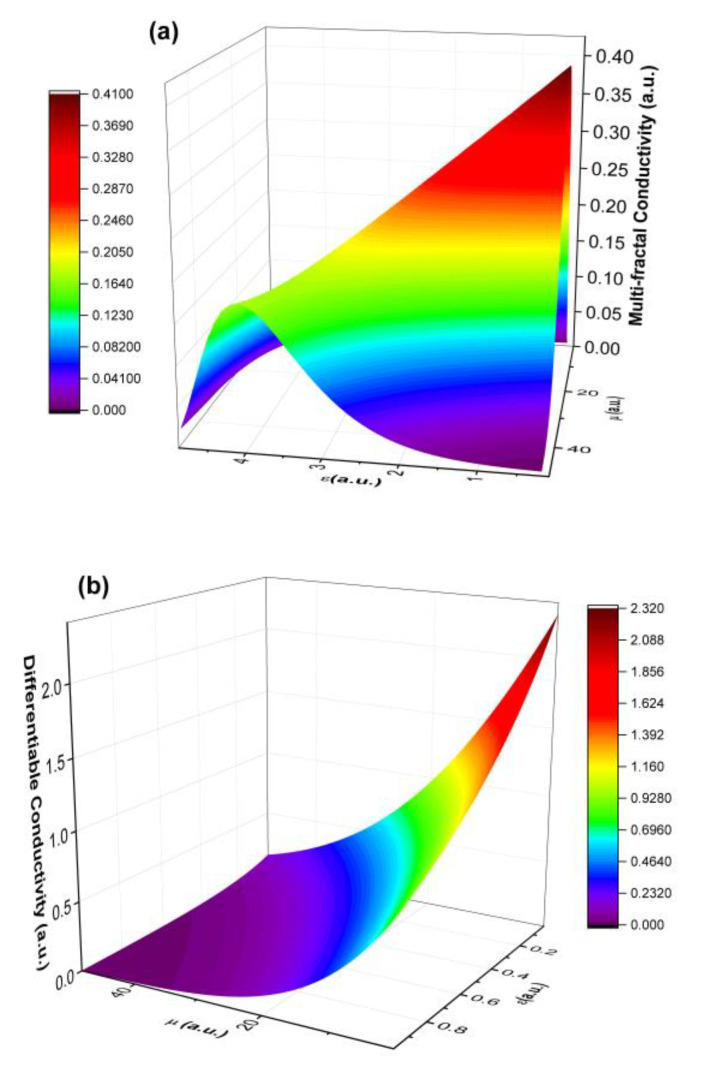
Three-dimensional representation of the two types of conductivities derived from the multifractal model.

We present in Figure 5a,b the theoretical dependencies of $\bar{\sigma_{D}}\left(\varepsilon \right)$ and $\bar{\sigma_{F}}\left(\varepsilon \right)$ for ξ,η=ct., and the restriction ξ≠η. By selecting clear resolution scales for particular types of conductivity, it was possible to address both various interaction scales and fractalization degrees. Conduction in complex systems is performed through specific mechanisms dependent on scale resolution. As a consequence, we made a distinction among differentiable conduction $\bar{\sigma_{D}}$, nondifferentiable conduction $\bar{\sigma_{F}}$, and global conduction $\bar{\sigma }$. Conduction mechanisms at the two types of scale resolutions are simultaneous and reciprocally conditional. Thus, the values of $\bar{\sigma_{D}}$ and $\bar{\sigma_{F}}$ increase along with increases in the ordering degree (synchronous type conductions), and with increases in the multifractalization degree, $\bar{\sigma_{D}}$ values increase and $\bar{\sigma_{F}}$ values decrease (asynchronous type conductions). We also noticed that higher degrees of multifractalization were seen as a higher mismatch in long scale orientation of the polymer. In the framework of the model, this read as losses in the inflection point of the trajectory. The conductivity of the polymer in the multifractal interpretations was seen as a measure of the available electron fluid to be transferred in different points of the material. The flow of the current was well characterized by the multifractal hydrodynamic model; thus, in each inflection point of the electron trajectories, losses could have appeared and thus led to lower conductivity. There was also an optimum at which we could obtain relatively higher conductivity. This point was unstable, as the system was overcome by losses and the conductivity decreased again. With decreases in the fractalization degree, we observed an exponential-type increase in conductivity.

**Figure 6:**
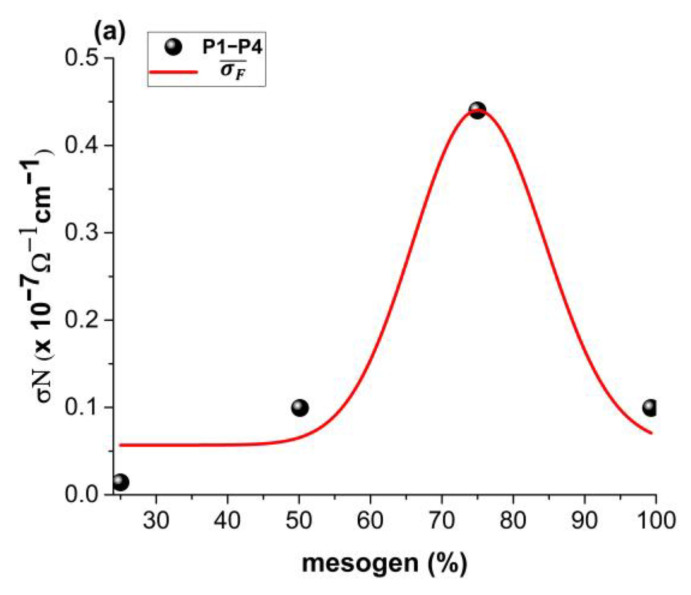

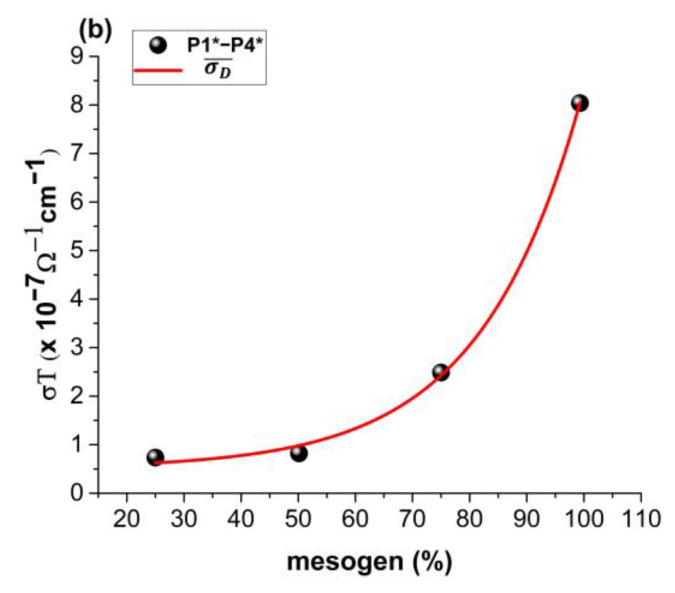
Calibration of the multifractal model on the untreated (**a**) and treated (**b**) polymers.

The validity of the model was tested by fitting the empirical data presented in Figure 6. The fit was performed setting a fix variable η=50, which was a high enough value to emulate long-time behavior, while all the other parameters were left unbound. From the fit, we extracted an average fractalization value at which each particular type of conductivity could be found. For fractal-type conductivity, an average of 0.3 was found, while for the differentiable type, an average of 5.1 was found.

## 4. Conclusions

The improvement of the electrical conductivity of thin films by thermal treatment into the mesophase state was investigated by creating a mathematical model based on a multifractal hydrodynamic model. The mathematical model predicted the possibility of two types of conductivity based on the fractalization degree of the system. The experimental measurements demonstrated that the electrical conductivity was significantly improved by thermal pretreatment, which contributed on one hand to the ordering of the mesogenic units into a continuous phase and on the other hand to jumping of electrons among the chains. The thermal pretreating also contributed to the nanostructuring of the surface, which constituted a good premise for bioapplications. The decrease in fractalization by thermal treatment changed the nature of the conductivity as defined by an exponential-type function. The validation of the model by fitting the empirical data revealed average fractalization values for which each type of conductivity was dominant.

## Data Availability

The data presented in this study are available on request from the corresponding authors.

## Supplementary Materials

The following supporting information can be downloaded at https://www.mdpi.com/article/10.3390/polym14071487/s1, Figure S1. Double logarithmic representation of roughness *versus* scan size of untreated (black lines) and treated (red lines) P1 (a), P2 (b), P3 (c) and P4 (d) film; Table S1: Average values of Ra and its standard deviation at scan size of 1000 nm × 1000 nm.

## References

[1] Bronstein H., Nielsen C.B., Schroeder B.C., McCulloch I. (2020). The role of chemical design in the performance of organic semiconductors. Nat. Rev. Chem..

[2] Bendrea A.D., Cianga L., Cianga I. (2011). Progress in the Field of Conducting Polymers for Tissue Engineering Applications. J. Biomat. Appl..

[3] Ramanavicius S., Ramanavicius A. (2021). Conducting Polymers in the Design of Biosensors and Biofuel Cells. Polymers.

[4] Lee S., Ozlu B., Eom T., Martin D.C., Shim B.S. (2020). Electrically conducting polymers for bio-interfacing electronics: From neural and cardiac interfaces to bone and artificial tissue biomaterials. Biosens. Bioelectron..

[5] Silva A.C.d., Torresi S.I.C.d. (2019). Advances in Conducting, Biodegradable and Biocompatible Copolymers for Biomedical Applications. Front. Mater..

[6] Li G., Yu K., Noordijk J., Meeusen-Wierts M.H., Gebben B., Lohuis P.A.M., Schotman A.H.M., Bernaerts K. (2020). Hydrothermal polymerization towards fully biobased polyazomethines. Chem. Commun..

[7] Lei T., Guan M., Liu J., Lin H.C., Pfattner R., Shaw L., McGu A.F. (2017). Biocompatible and totally disintegrable semiconducting polymer for ultrathin and ultralightweight transient electronics. Proc. Natl. Acad. Sci. USA.

[8] Bolduc A., Ouahabi A.A., Mallet C., Skene W.G. (2013). Insight into the isoelectronic character of azomethines and vinylenes using representative models—A spectroscopic and electrochemical study. J. Org. Chem..

[9] Zabulica A., Balan M., Belei D., Sava M., Simionescu B.C., Marin L. (2013). Novel luminescent phenothiazine-based Schiff bases with tuned morphology. Synthesis, structure, photophysical and thermotropic characterization. Dye. Pigment..

[10] Katariya K.D., Nakum K.J., Hagar M. (2021). New fluorinated azo/Schiff base liquid crystals: Synthesis, characterization, mesomorphic study and DFT calculations. Liq. Cryst..

[11] He Q.Q., Lan Y., Quan Y.Y., Li C.Y., Liu Y.P., Wang X.J., Jia Y.G., Tian M., Yao D.S. (2021). The influence of the structure of terminal groups and cores on the properties of schiff base star-shaped liquid crystals. Liq. Cryst..

[12] Zhang L., Zhao K., Li H., Zhang T., Liu D., Han Y. (2019). Liquid crystal ordering on conjugated polymers film morphology for high performance. J. Polym. Science Polym. Phys..

[13] Marin L., Timpu D., Cozan V., Rusu G.I., Airinei A. (2011). Solid State Properties of Thin Films of New Copoly(azomethine-sulfone)s. J. Appl. Polym. Sci..

[14] Ciobanu M., Cozan V., Bruma M. (2009). Aromatic polysulfones used in sensor applications. Rev. Adv. Mat. Sci..

[15] Jiang Q., Zhang Q., Wu X., Wu L., Lin J.H. (2020). Exploring the Interfacial Phase and π–π Stacking in Aligned Carbon Nanotube/Polyimide Nanocomposites. Nanomaterials.

[16] Li J.-L., Yin J.-H., Ji T., Feng Y., Liu Y.-Y., Zhao H., Li Y.-P., Zhu C., Yue D., Su B. (2020). Microstructure evolution effect on high-temperature thermal conductivity of LDPE/BNNS investigated by in-situ SAXS. Mat. Lett..

[17] Mombrú D., Romero M., Faccio R., Castiglioni J., Mombrú A.W. (2017). In situ growth of ceramic quantum dots in polyaniline host via water vapor flow diffusion as potential electrode materials for energy applications. J. Solid State Chem..

[18] Khan I., Mohan S.D., Belbut M., Kamma-Lorger C.S., Mateus A., Mitchell G.R. (2017). Multiscale structure evolution in electrically conductive nanocomposites studied by SAXS. Proc. Manufact..

[19] Ailincai D., Dorobanțu A.M., Dima B., Irimiciuc S.A., Lupașcu C., Agop M., Orzan O. (2020). Poly(vinyl alcohol boric acid)-Diclofenac Sodium Salt Drug Delivery Systems: Experimental and Theoretical Studies. J. Immun. Res..

[20] Ailincai D., Agop M., Marinas I.C., Zala A., Irimiciuc S.A., Dobreci L., Petrescu T.C., Volovat C. (2021). Theoretical model for the diclofenac release from PEGylated chitosan hydrogels. Drug Deliv..

[21] Iftime M.M., Irimiciuc S.A., Agop M., Angheloiu M., Ochiuz L., Vasincu D. (2020). A Theoretical Multifractal Model for Assessing Urea Release from Chitosan Based Formulations. Polymers.

[22] Destri S., Porzio W., Bertini F. (2009). Synthesis and characterization of new azomethine derivatives exhibiting liquid crystalline properties. Liq. Cryst..

[23] Sivaranjini B., Umadevi S., Khan R.K., Pratibha R., Dekshinamoorthy A., Vijayaraghavan S., Ganesh V. (2021). Planar and Vertical Alignment of Rod-like and Bent-core Liquid Crystals Using Functionalized Indium Tin Oxide Substrates. Liq. Cryst..

[24] Manabe A., Bremer M., Kraska M. (2021). Ferroelectric nematic phase at and below room temperature. Liq. Cryst..

[25] Marin L., Bejan A., Ailincai D., Belei D. (2017). Poly(azomethine-phenothiazine)s with efficient emission in solid state. Eur. Polym. J..

[26] Macanas J., Palacio L., Pradanos P., Hernandez A., Munoz M. (2006). Atomic force microscopy as a suitable technique for surface characterization of activated composite membranes for metal ion facilitated transport. Appl. Phys. A..

[27] Tan D.Q. (2020). Differentiation of roughness and surface defect impact on dielectric strength of polymeric thin films. IET Nanodielectr..

[28] Popovici D., Diaconu A., Rotaru A., Marin L. (2019). Microwave-Assisted Synthesis of an Alternant Poly(fluorene-oxadiazole). Synthesis, Properties, and White Light-Emitting Devices. Polymers.

[29] Wang C., Dong H., Jiang L., Hu W. (2018). Organic semiconductor crystals. Chem. Soc. Rev..

[30] Nasrin L., Nasir A.K., Yoshizawa A., Ghosh S., Rahman M. (2019). Nematic—cybotactic nematic phase transition in a liquid crystal: A dielectric spectroscopic study. Mater. Res. Express.

[31] Marin L., Zabulica A., Sava M. (2013). Symmetric Liquid Crystal Dimers Containing a Luminescent Mesogen: Synthesis, Mesomorphic Behavior, and Optical Properties. Soft Mater..

[32] Schliemann J. (2006). Ballistic side jump motion of electrons and holes in semiconductor quantum wells. Phy. Rev. B Cond. Matt..

[33] Cobzeanu B.M., Irimiciuc S., Vaideanu D., Grigorovici A., Popa O. (2017). Possible dynamics of polymer chains by means of a Ricatti’s procedure-an exploitation for drug release at large time intervals. Mat. Plast..

[34] Agop M., Mercheș I. (2019). Operational Procedures Describing Physical Systems.

[35] Merches I., Agop M. (2016). Differentiability and Fractality in Dynamics of Physical Systems.

[36] Agop M., Păun V.P. (2017). On the New Perspectives of Fractal Theory: Fundaments and Application.

