# Supplementary material for: Impact of the Liquid Crystal Order of Poly(azomethine-sulfone)s on the Semiconducting Properties
Source: Polymers (Basel). 2022 Apr 6;14(7):1487. doi: 10.3390/polym14071487 (PMC9003125; doi:10.3390/polym14071487)
Supplement: Supplementary file 1 [file polymers-14-01487-s001.zip › polymers-1650213-supplementary.pdf]

polymers

MDPI

Supporting Information for

Impact of the Liquid Crystal Order of Poly(Azomethine-Sulfone)s on the Semiconducting Properties

Oana Dumbravă [1], Dumitru Popovici [1], Decebal Vasincu [2], Ovidiu Popa [3], Lăcrămioara Ochiuz [4], Ștefan-Andrei Irimiciuc [5,]*, Maricel Agop [6,7,]* and Anca Negură [8]

1 "Petru Poni" Institute of Macromolecular Chemistry, Gr. Ghica Voda Alley, 41A, 700487 Iasi, Romania; dumbrava.oana@icmpp.ro (O.D.); dumitru.popovici@icmpp.ro (D.P.)

2 Department of Biophysics and Medical Physics, Faculty of Dental Medicine, "Grigore T. Popa" University of Medicine and Pharmacy, 16 University Str., 700115 Iasi, Romania; decebal.vasincu@umfiasi.ro

3 Department of Emergency Medicine, Faculty of Medicine, "Grigore T. Popa" University of Medicine and Pharmacy, 16 University Str., 700115 Iasi, Romania; ovidiu.popa@umfiasi.ro

4 Department of Pharmaceutical Sciences, Faculty of Pharmacy, "Grigore T. Popa" University of Medicine and Pharmacy, 16 University Str., 700115 Iasi, Romania; lacramioara.ochiuz@umfiasi.ro

5 National Institute for Laser, Plasma and Radiation Physics, 409 Atomistilor Street, 077125 Bucharest, Romania

6 Department of Physics, "Gh. Asachi" Technical University of Iasi, 700050 Iasi, Romania

7 Romanian Scientists Academy, 54 Splaiul Independentei, 050094 Bucharest, Romania

8 Faculty of Biology, "Alexandru Ioan Cuza" University of Iasi, 2A Carol Boulevard, 700505 Iasi, Romania; anca.negura@uaic.ro

* Correspondence: stefan.irimiciuc@inflpr.ro (Ș.-A.I.); magop@tuiasi.ro (M.A.)

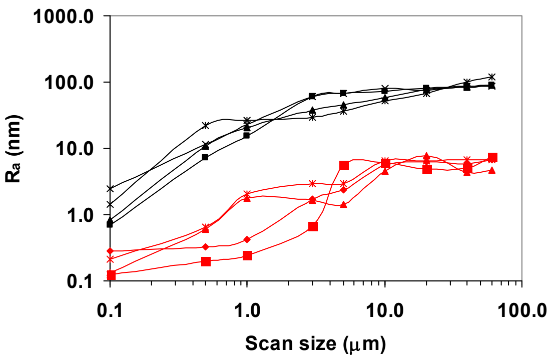

(**a**) P1

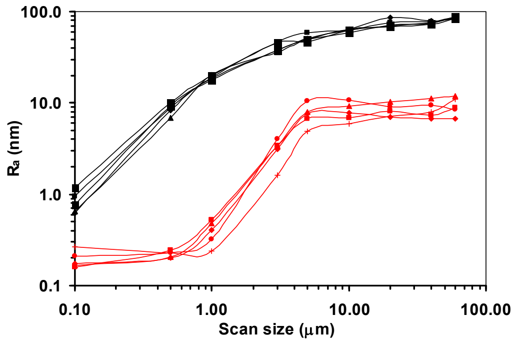

(**b**) P2

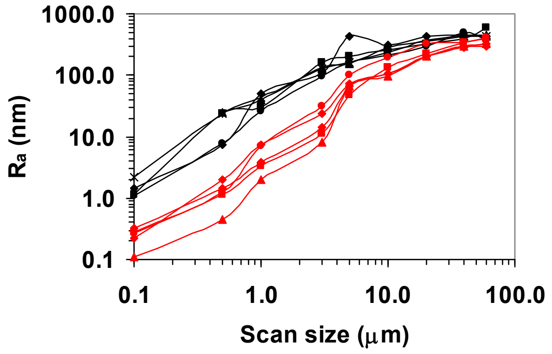

(**c**) P3

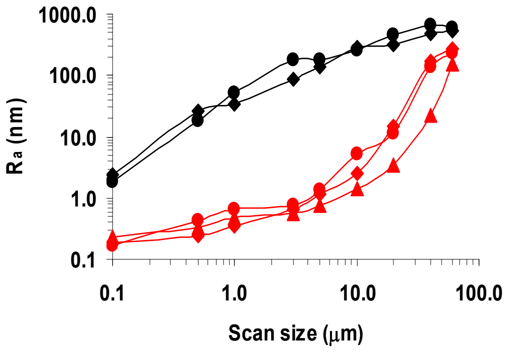

(**d**) P4

Figure S1. Double logarithmic representation of roughness *versus* scan size of untreated (black lines) and treated (red lines) P1(a), P2 (b), P3(c) and P4 (d) film.

Table S1. Average values of Ra and its standard deviation at scan size of 1000 nm x 1000 nm.

**Sample Code**	**P1**	**P1***	**P2**	**P2***	**P3**	**P3***	**P4**	**P4***
**Ra(nm)**	20.25 ± 2.0	1.10 ± 0.4	17.64 ± 0.8	0.38 ± 0.1	37.0 ± 3.9	4.27 ± 1.0	39.53 ± 4.6	0.45 ± 0.1
